# VEGF-D Serum Level as a Potential Predictor of Lymph Node Metastasis and Prognosis in Vulvar Squamous Cell Carcinoma Patients

**DOI:** 10.3389/fonc.2022.818613

**Published:** 2022-04-08

**Authors:** Antonella Ravaggi, Angela Gambino, Federico Ferrari, Alessandro Olivari, Laura Zanotti, Chiara Romani, Laura Ardighieri, Paolo Antonelli, Giorgia Garganese, Daniela Gallo, Giovanni Scambia, Eliana Bignotti, Enrico Sartori, Stefano Calza, Franco Odicino

**Affiliations:** ^1^ Department of Clinical and Experimental Sciences, Division of Obstetrics and Gynecology, University of Brescia, Brescia, Italy; ^2^ Division of Obstetrics and Gynecology, Azienda Socio Sanitaria Territoriale Spedali Civili di Brescia, Brescia, Italy; ^3^ ’Angelo Nocivelli’ Institute of Molecular Medicine, University of Brescia—Azienda Socio Sanitaria Territoriale Spedali Civili, Brescia, Italy; ^4^ Department of Pathology, Azienda Socio Sanitaria Territoriale Spedali Civili di Brescia, Brescia, Italy; ^5^ Unit of Biostatistics and Bioinformatics, Department of Molecular and Translational Medicine, University of Brescia, Brescia, Italy; ^6^ Gynecology and Breast Care Center, Mater Olbia Hospital, Olbia, Italy; ^7^ Dipartimento Universitario Scienze della Vita e Sanità Pubblica, Sezione di Ginecologia ed Ostetricia, Università Cattolica del Sacro Cuore, Rome, Italy; ^8^ Dipartimento Scienze della Salute della Donna, del Bambino e di Sanità Pubblica, Fondazione Policlinico Universitario A. Gemelli, Istituto di Ricovero e Cura a Carattere Scientifico, Rome, Italy; ^9^ BDbiomed, Big & Open Data Innovation Laboratory, University of Brescia, Brescia, Italy

**Keywords:** vulvar squamous cell carcinoma, VEGF-D, lymph node metastasis, prognosis, serum

## Abstract

**Background:**

Radical surgical resection of the primary tumor with mono/bilateral inguinofemoral lymph node dissection is the standard treatment for invasive vulvar squamous cell carcinoma (VSCC) and is frequently related to severe morbidity. Tailoring surgical treatment is of paramount importance, and a comprehensive preoperative evaluation is mandatory. Vascular endothelial growth factor D (VEGF-D) is considered a regulator of lymphangiogenesis involved in tumor spread *via* lymphatic vessels. The aim of this study was to evaluate the potential of VEGF-D in the prediction of inguinofemoral lymph node metastasis.

**Methods:**

We analyzed the preoperative levels of serum VEGF-D (sVEGF-D) from two independent cohorts of patients with VSCC by enzyme-linked immunosorbent assay and its protein expression on tumor tissue by immunohistochemistry. Logistic regression was performed to identify the independent risk factors for lymph node metastasis, and Cox proportional hazard model was used for survival analysis.

**Results:**

High levels of sVEGF-D, but not tissue VEGF-D, significantly correlated with positive groin nodes and a more advanced International Federation of Gynecologists and Obstetricians (FIGO) stage. In multivariable analysis, a high sVEGF-D level was an independent predictor of lymph node metastasis and worse prognosis. A prediction model based on sVEGF-D, tumor grade assessed on biopsy, tumor diameter, and lymph node clinical evaluation was able to predict lymph node metastasis, reaching C-index values of 0.79 and 0.73 in the training and validation cohorts, respectively.

**Conclusions:**

The preoperative sVEGF-D level might be a reliable biomarker for the prediction of lymph node metastasis and prognosis in patients with VSCC, supporting better clinical/surgical decision. Multicenter prospective studies are required to confirm our findings.

## Introduction

Vulvar carcinoma is a rare gynecologic cancer with an annual incidence of 2.4 every 100,000 women based on cases in 2014–2018 (1). In 2021, approximately 6,120 new cases of vulvar cancer have been predicted in the United States, and about 1,550 women are expected to die of this cause (2). The incidence is higher among women aged 70 years or older; however, recently, an increasing frequency of vulvar cancer in young women has been observed (3, 4).

For patients with invasive vulvar squamous cell carcinoma (VSCC), the most common type of vulvar cancer, the standard treatment in clinical early-stage disease is radical resection of the tumor with bilateral inguinofemoral lymphadenectomy or sentinel lymph node (SLN) biopsy, when appropriate. In the absence of clinical suspicious lymph nodes (cN0), the SLN procedure should be considered the preferred procedure instead of radical dissection only within narrow selection criteria, including: unifocal lesions of <4 cm, not completely excised at diagnostic biopsy, and never undergone previous vulvar/groin surgery or neoadjuvant treatments (5–7). Furthermore, the SLN procedure should be performed only in centers with high-level experience in order to minimize false-negative rates. Therefore, a large cohort of cN0 patients is currently not submitted to the SLN procedure and instead referred to radical lymphadenectomy. This procedure showed no evidence of lymph node metastasis at final histology in 70% of cases, with increased postoperative physical and psychological morbidity rates (8). For this reason, many efforts are being made to optimize clinical management in order to avoid overtreatment of patients without lymph node metastasis and to ensure a high diagnostic sensitivity in identifying patients with positive groin nodes (9).

In this context, the identification of circulating or tissue biomarkers involved in the aggressiveness of tumor cells and in the metastasis process could be an important tool to guide and/or refine the surgical decision.

One of the most studied molecular systems in the regulation of tumor lymphangiogenesis is based on the interaction between vascular endothelial growth factor D (VEGF-D) and the corresponding VEGF receptor 3 (VEGFR-3), commonly expressed on the surface of lymphatic endothelial cells (10, 11). VEGF-D is a member of the VEGF family, which also includes VEGF-A, VEGF-B, VEGF-C, and placental growth factor (PlGF), and it is an important key regulator of both physiological and pathological angiogenesis and lymphangiogenesis (11). VEGF-D promotes lymphatic metastasis by inducing tumor-associated lymphangiogenesis in a mouse tumor model (12), and its over expression was associated with lymphatic tumor spread and poor patient prognosis in several human cancers (13–16). To the best of our knowledge, only one study evaluated the expressions of VEGF-D and VEGFR-3 using immunohistochemistry (IHC) in VSCC (17). Further investigations are required to clarify the clinical significance of these markers in patients with VSCC.

In the present study, we investigated the role of VEGF-D in predicting inguinofemoral lymph node metastasis. For the first time, the preoperative levels of serum VEGF-D (sVEGF-D) were quantified and correlated with the clinicopathological characteristics and prognosis of patients affected by VSCC.

## Methods

### Study Design

This retrospective study aimed to investigate the significance of VEGF-D in the clinical setting of VSCC patients and, in particular, in the preoperative prediction of lymph node metastasis. To this intent, two groups of patients with VSCC were recruited. Cohort A was used to analyze the correlations between sVEGF-D levels or the tissue expressions of VEGF-D and VEGFR-3 with the clinicopathological features and prognosis. Moreover, more importantly, cohort A was used as a training set to design a predictive algorithm for lymph node metastases. Three different models for the preoperative prediction of lymph node involvement were built using logistic regression: *base model*, including only clinical/radiological lymph node status; *clinical model*, including the clinicopathological characteristics available before surgery (e.g., tumor diameter, tumor grade from biopsy, and clinical lymph node status); and *extended model*, built with the addition of the sVEGF-D level to the clinical model.

Cohort B was kept external and independent and was used as a validation set to evaluate the performance of the extended model in the prediction of lymph node metastasis.

### Patients and Samples

A retrospective study was performed on a total of 135 patients with VSCC divided into two independent cohorts, hereafter called A and B, comprising 80 and 55 women, respectively.

The inclusion criteria were as follows: histologically confirmed VSCC, tumor depth of invasion of at least 1 mm, surgical treatment performed in the enrolling centers, availability of frozen serum samples collected prior to surgery, and/or availability of formalin-fixed paraffin-embedded (FFPE) tumor tissue samples surgically obtained from the primary tumor site. For cohort B, only serum samples were required. Patients with synchronous cancer or with a history of malignancy in the 5 years prior to the VSCC diagnosis were excluded from the study of circulating sVEGF-D.

The eligible patients of cohort A were consecutively enrolled between January 2003 and August 2019 at the Division of Obstetrics and Gynecology of “ASST-Spedali Civili di Brescia” (Brescia, Italy). The eligible patients of cohort B were consecutively enrolled between May 2012 and October 2018 at the Division of Gynecologic Oncology of “Fondazione Policlinico Universitario A. Gemelli IRCCS” (Rome, Italy).

All patients were preoperatively assessed by expert gynecologic oncologists and dedicated specialists for primary tumor histology, obtained by incisional biopsy, and lymph node status evaluation, obtained by clinical exam and dedicated imaging (ultrasound and/or PET). A positive clinical lymph node status was defined in the presence of at least one suspicious lymph node at imaging.

All patients were surgically treated by complete surgical tumor resection (partial or radical vulvectomy) and inguinal lymph node dissection, performed mono- or bilaterally, as appropriate, on the base of the distance of the primary tumor from the midline, according to international guidelines (5, 6) at the time of the study.

All 80 patients from cohort A underwent radical lymphadenectomy. Among the 55 patients in cohort B, 12 underwent exclusive SLN, 9 underwent combined SLN and inguinofemoral lymphadenectomy, and 34 underwent inguinofemoral lymphadenectomy.

The stage of disease was assessed in accordance with the International Federation of Gynecologists and Obstetricians (FIGO) revised staging system of 2009, in use during the enrollment period.

Adjuvant treatment was administered to 24 out of 80 patients in cohort A (radiotherapy, chemotherapy, or chemoradiotherapy to 21, 2, and 1 patient, respectively). Fifty-one patients did not receive adjuvant therapies, and for 5 patients, this information was missing. All patients in cohort A were followed from the time of their confirmed diagnosis until death or March 2021. Follow-up data were not required for the patients in cohort B. Clinical and histopathological data were acquired from the original reports.

The characteristics of the patients in the two cohorts are summarized in **Table 1**.

**Table 1:** Clinical characteristics of vulvar squamous cell carcinoma (VSCC) patients.

Clinical annotations	Cohort A	Cohort B	Total
**No. of patients**	80	55	135
**Age (years)**			
Mean (SD)	70.2 (11.7)	71.4 (11.4)	70.7 (11.5)
Median (Q1–Q3)	70.5 (63.0–79.0)	74.0 (64.0–80.0)	73.0 (63.0–80.0)
**Clinical lymph node status**			
Negative	43 (60.6%)	28 (50.9%)	71 (56.3%)
Positive	28 (39.4%)	27 (49.1%)	55 (43.7%)
Missing	9	0	9
**Tumor grade on biopsy**			
G1	30 (38.5%)	12 (30.8%)	42 (35.9%)
G2–G3	48 (61.5%)	27 (69.2%)	75 (64.1%)
Missing	2	16	18
**Diameter (mm)**			
Mean (SD)	34.5 (17.5)	37.8 (20.2)	35.8 (18.6)
Median (Q1–Q3)	30.0 (22.0–45.8)	32.5 (19.5–57.0)	30 (20.0–50.0)
Missing	0	1	
**FIGO stage**			
I	43 (53.8%)	28 (50.9%)	71 (52.6%)
II	3 (3.7%)	2 (3.6%)	5 (3.7%)
III	33 (41.3%)	23 (41.8%)	56 (41.5%)
IV	1 (1.2%)	2 (3.7%)	3 (2.2%)
**Tumor grade**			
G1	18 (22.5%)	4 (7.3%)	22 (16.3%)
G2	50 (62.5%)	40 (72.7%)	90 (66.7%)
G3	12 (15.0%)	11 (20.0%)	23 (17.0%)
**Depth of invasion (mm)**			
Mean (SD)	9.9 (8.5)	7.8 (5.1)	9.0 (7.4)
Median (Q1–Q3)	8.0 (5.0–13.0)	6.0 (4.0–10.5)	7.0 (4.0–12.0)
Missing	1	2	3
**Vascular invasion**			
Absent	57 (71.3%)	29 (61.7%)	86 (67.7%)
Present	23 (28.7%)	18 (38.3%)	41 (32.3%)
Missing	0	8	8
**Perineural invasion**			
Absent	49 (62.0%)	42 (87.5%)	91 (71.7%)
Present	30 (38.0%)	6 (12.5%)	36 (28.3%)
Missing	1	7	8
**Lymph node metastasis**			
Absent	46 (57.5%)	30 (54.5%)	76 (56.3%)
Present	34 (42.5%)	25 (45.5%)	59 (43.7%)
**Surgical margins**			
Negative	65 (82.3%)	52 (94.5%)	117 (87.3%)
Positive	14 (17.7%)	3 (5.5%)	17 (12.7%)
Missing	1	0	1

Q1, first quartile; Q3, third quartile; SD, standard deviation.

The study was performed following the set of principles in the Declaration of Helsinki and was approved by the Research Review Board–Ethics Committee of the ASST Spedali Civili, Brescia, Italy (study reference no. NP3512) and of the Fondazione Policlinico Universitario “A. Gemelli” IRCCS—Università Cattolica del Sacro Cuore, Rome, Italy (study reference no. ID2844). Written informed consent for the collection and use of personal records and biological material for health research was obtained from all patients enrolled. All data were collected in an electronic database and managed in accordance with privacy regulations.

### VEGF-D Serum Concentration Measurement

In both cohorts, fasting blood samples were drawn from patients strictly before surgery. Serum was separated by centrifugation at 1,500 × *g* for 10 min within 1 h, frozen in liquid nitrogen, and then stored at −80°C until analysis. Sample analysis was centralized in Brescia Hospital laboratory and carried out without any prior knowledge of the patients’ clinical status.

The sVEGF-D levels were analyzed with enzyme-linked immunosorbent assay (ELISA) using the immunoassay Human VEGF-D Quantikine ELISA (R&D Systems, Minneapolis, MN, USA) following the manufacturer’s instructions. Briefly, 50 ml of standards, controls, and serum samples were analyzed in duplicate, and plates were read at 450 nm, setting a wavelength correction to 570 nm, on an automatic plate reader (Spectramax 340 PC; Molecular Devices Corporation, Sunnyvale, CA, USA). As declared by the manufacturer, the dynamic range of VEGF-D detection goes from 125 to 4,000 pg/ml, with intra-assay and inter-assay imprecision (coefficient of variation, CV) values ranging from 2.4% to 6.2% and from 7.2% to 8.0%, respectively.

### Immunohistochemical Staining of VEGF-D and VEGFR-3 on Formalin-Fixed Tumor Tissues

To evaluate the protein expression levels of VEGF-D and VEGFR-3, IHC staining was performed on VSCC tissue samples from cohort A at the Department of Pathology, “ASST-Spedali Civili of Brescia.” Whole tissue sections (2 μm) were obtained from FFPE blocks, stained with hematoxylin and eosin, and analyzed by a staff surgical pathologist. IHC analyses were performed on 4-μm tissue sections using the Leica Bond III fully automated IHC Stainer (Leica Biosystems, New Castle upon Tyne, UK). No antigen retrieval was carried out. The sections were incubated with the primary antibodies anti-VEGF-D diluted 1:200 (clone 78923; R&D Systems) for 15 min and anti-VEGFR-3 diluted 1:50 (clone NCL-L-VEGFR-3; Leica Biosystems) for 30 min. The reaction was revealed using the automated Leica BOND system by the Bond Polymer Refine Detection Kit (DS9800; Leica Biosystems), which consisted of sequential incubation with post-primary and horseradish peroxidase (HRP)–polymer for 8 min each, followed by diaminobenzidine as chromogen and by hematoxylin as nuclear counterstain.

Cellular staining was graded for intensity (0, negative staining; 1, weak staining; 2, moderate staining; and 3, strong staining) and percentage of positive cells (0, 0%; 1, 1%–20%; 2, 11%–50%; and 3, 51%–100%). A single IHC scale with scores 0–9 was calculated by multiplying the intensity and the percentage staining scores. Then, four total scores (0, 1, 2, 3) were obtained, grouping score 0 in total score 0, scores 1–3 in total score 1, scores 4 and 6 in total score 2, and score 9 in total score 3.

### Statistical Methods

The association between the levels of sVEGF-D and the clinicopathological characteristics in cohort A was evaluated using univariable linear models after transforming the sVEGF-D levels on a log_2_ scale.

The role of sVEGF-D as a predictor of lymph node involvement was evaluated using a logistic regression model including the clinicopathological characteristics available before radical vulvectomy: estimated tumor diameter, tumor grade assessed on biopsy, and lymph node status from clinical/radiological evaluation. The results were reported as odds ratios (ORs) and 95% confidence intervals (95%CIs).

The discrimination performance of the models was quantified with the concordance index (C-index), which is equivalent to the area under the receiver operating characteristic (ROC) curve (AUC). The 95%CI for the C-index was obtained after 200 bootstrap resampling (18). Model calibration was evaluated graphically and reporting scaled Brier scores (the bigger the better) (19) and the Hosmer–Lemeshow test (20). Unreliability test (testing the *H*_0_ for a calibration line with slope = 1 and intercept = 0) was performed using a chi-squared test with 2 *df* (21).

Penalized maximum likelihood was used to obtain more stable coefficients through shrinkage in order to achieve better performance for prediction (22, 23). Selection of the optimal penalization parameter was performed based on Hurvich and Tsai’s corrected Akaike’s information criterion (AIC) (24).

Progression-free survival (PFS) was defined as the time from surgery to progression or recurrence, while disease-specific survival (DSS) was defined as the time from surgery to cancer-related death. Univariable and multivariable Cox proportional hazard models were used for modeling PFS and DSS.

For display purposes, the sVEGF-D values were dichotomized using maximally selected rank statistics (25), both for DSS and PFS. Briefly, this procedure searches for the optimal threshold that maximizes the log-rank statistic, accounting for test multiplicity. Binary-coded sVEGF-D values were used in the multivariable models accounting for stage, grade, and vascular and perineural invasion, and the corresponding survival curves were displayed using the Kaplan–Meier method.

All tests were two-sided and assumed a 5% significance level. All statistical analyses were performed with the program R Core Team (version 4.1.1).

## Results

### VEGF-D Serum Level Correlates With Lymph Node Metastasis and FIGO Stage

Preoperative serum samples from 62 out of 80 patients in cohort A were suitable to analyze the sVEGF-D levels using ELISA. The median and the mean values of sVEGF-D were 456.0 pg/ml (range = 370.9–573.5) and 483.4 pg/ml (SD = 178.5), respectively. As shown in **Table 2**, high levels of sVEGF-D significantly correlated with lymph node metastasis (*p* = 0.023) and higher FIGO stage (III–IV *vs*. I–II, *p* = 0.023). No other significant correlations with the clinicopathological factors were evident.

**Table 2:** Vascular endothelial growth factor D (VEGF-D) serum levels and correlation with the clinicopathological characteristics of vulvar squamous cell carcinoma (VSCC) patients from cohort A (*N* = 62).

Characteristics	Levels (*N*)	sVEGF-D (pg/ml)			
		Mean (SD)	Median	Q1–Q3	*p*-value^a^
Age (years)	<72 (31)	485.3 (116.4)	474.8	406.8–558.9	0.933
	≥72 (31)	481.4 (226.3)	408.9	344.8–604.8	
FIGO stage	I–II (36)	440.0 (135.1)	420.2	360.3–542.3	**0.023**
	III–IV (26)	543.4 (213.9)	476.9	410.1–666.7	
Tumor grade	G1 (16)	490.8 (259.9)	436.8	310.0–601.1	0.435
	G2 (38)	465.2 (146.8)	428.8	374.7–534.5	
	G3 (8)	554.7 (106.5)	527.2	477.5–598.6	
Tumor diameter (mm)	≤20 (16)	495.8 (141.2)	471.8	414.0–586.8	0.428
	21–40 (26)	449.6 (133.9)	428.8	355.3–564.6	
	>40 (20)	517.3 (245.1)	465.1	382.5–571.0	
Depth of invasion (mm)	≤8 (29)	489.9 (149.3)	482.8	383.2–615.9	0.771
	>8 (32)	476.3 (206.0)	429	368.5–493.2	
	NA (1)				
Vascular invasion	No (43)	491.7 (193.8)	456.7	372.2–574.5	0.587
	Yes (19)	464.6 (140.9)	455.4	378.6–528.4	
Perineural invasion	No (40)	492.0 (203.4)	464.8	360.3–591.1	0.572
	Yes (21)	464.3 (125.7)	438.4	386.3–518.6	
	NA (1)				
Lymph node metastasis	No (36)	440.0 (135.1)	420.2	360.3–542.3	**0.023**
	Yes (26)	543.4 (213.9)	476.9	410.1–666.7	
Recurrence	No (29)	444.6 (167.6)	438.4	320.8–540.6	0.115
	Yes (32)	517.4 (186.3)	464.1	392.3–618.6	
	NA (1)				
Adjuvant treatment	No (42)	461.3 (158.3)	429.8	363.7–545.8	0.465
	Yes (18)	492.8 (136.2)	465.1	405.3–574.2	
	NA (2)				

Significant p-values are indicated in bold.

FIGO, International Federation of Gynecologists and Obstetricians; Q1, first quartile; Q3, third quartile; NA, not available.

a Linear model ANOVA on log2-transformed sVEGF-D values.

In 49 out of 80 patients, FFPE tumor tissue samples surgically obtained from the primary tumor site were available to evaluate the tissue protein expression of VEGF-D and its receptor VEGFR-3. A pattern of positive cytoplasmic expression for VEGF-D and VEGFR-3 was found in 92% and 94% of the VSCC patients, respectively (**Supplementary Figure S1**). No correlation was found between the expression of VEGF-D or VEGFR-3 and the clinicopathological characteristics of the tumor (**Supplementary Table S1**); therefore, the next steps of the study were conducted considering exclusively serum VEGF-D.

For 31 out of 80 patients, matched tissue and serum samples were available. The serum VEGF-D level was only partially correlated with the tissue VEGF-D protein score, with a borderline statistical significance (*r*_s_ = 0.317, *p* = 0.082).

### High Level of sVEGF-D Correlates With Poor Prognosis

In cohort A, 79 out of 80 patients were considered for survival analysis (median follow-up = 101.1 months, IQR = 58.0–150.5 months). Forty-one (51.9%) had disease recurrence or progression. At the time of the last follow-up, 36 patients (45.6%) were alive, 30 (38.0%) were dead of disease, and 13 (16.4%) died of other causes.

**Figure 1:**
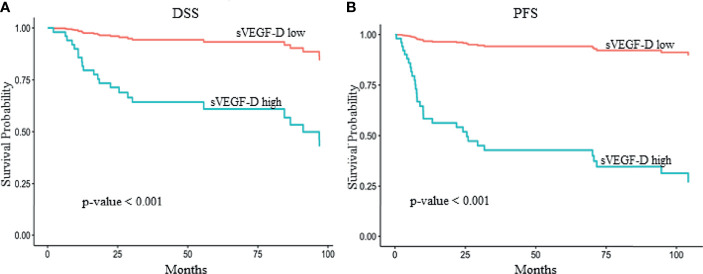
Kaplan–Meier survival curves showing the effect of serum vascular endothelial growth factor D (sVEGF-D) level in multivariate models adjusted for stage, grade, and vascular and perineural invasion. The optimal threshold for sVEGF-D categorization was determined by maximally selected rank statistics. The reported *p*-values were adjusted for multiple testing. Higher sVEGF-D levels [>393 and >329 pg/ml for disease-specific survival (DSS) and progression-free survival (PFS), respectively] exhibited a significant association with reduced DSS **(A)** (*p* < 0.001) and PFS **(B)** (*p* < 0.001) in 61 patients with vulvar squamous cell carcinoma (VSCC) from cohort A.

In the univariable analysis for DSS, higher preoperative sVEGF-D levels (log scale) were significantly associated with worse prognosis [hazard ratio (HR) = 2.70, *p* = 0.02] and with other traditional prognostic factors, such as advanced FIGO stage (HR = 9.43, *p* < 0.01), perineural invasion (HR = 2.17, *p* = 0.03), lymphovascular invasion (HR = 2.55, *p* = 0.01), and lymph node metastasis (HR = 9.43, *p* < 0.01) (**Figure 1A** and **Supplementary Table S2**).

In addition, in the univariable analysis for PFS, higher preoperative sVEGF-D levels (HR = 2.15, *p* = 0.036), lymph node metastasis (HR = 3.20, *p* < 0.01), lymphovascular invasion (HR = 1.95, *p* = 0.04), and advanced FIGO stage (HR = 3.20, *p* < 0.01) were significantly associated with a high risk of recurrence (**Figure 1B** and **Supplementary Table S2**).

In the multivariable analysis, the preoperative sVEGF-D levels (entered as a restricted cubic spline with 3 knots, i.e., a quadratic trend) were demonstrated to be independent prognostic factors for poor DSS (likelihood ratio test: $\chi_{2}^{2}=7.30,$ *p* = 0.026) (**Table 3** and **Figure 1A**). Similarly, in the multivariable analysis for PFS, sVEGF-D (linear trend) was marginally significant (*p* = 0.047) (**Table 3** and **Figure 1B**).

**Table 3:** Multivariable survival analysis for both disease-specific survival (DSS) and progression-free survival (PFS) on 61 VSCC patients from cohort A.

Variables	DSS		PFS	
	HR (95%CI)	*p*-value	HR (95%CI)	*p*-value
FIGO stage				
III–IV *vs*. I–II	10.5 (3.28–33.55)	**<0.001**	2.25 (0.99–5.14)	0.053
Tumor grade				
G2–G3 *vs*. G1	0.68 (0.20–2.30)	0.540	0.46 (0.19–1.15)	0.098
Vascular invasion				
Yes *vs*. no	1.88 (0.68–5.20)	0.220	2.08 (0.84–5.15)	0.116
Perineural invasion				
Yes *vs*. no	1.63 (0.56–4.75)	0.370	1.58 (0.62–4.01)	0.339
sVEGF-D				
Q3 *vs*. Q2	3.15 (1.21–8.23)	**0.026^a^**	1.86 (1.09–3.17)	**0.047^a^**

Significant p-values are indicated in bold.

HR, hazard ratio; FIGO, International Federation of Gynecologists and Obstetricians; sVEGF-D, serum vascular endothelial growth factor D; Q2, second quartile; Q3, third quartile.

**^a^** Likelihood ratio test-based p-value.

### Association Between Lymph Node Metastasis and Clinicopathological Characteristics

In order to assess the factors associated with lymph node metastasis and to build a predictive algorithm, pathological lymph node status was compared with the clinicopathological variables available before and after surgery. As reported in **Supplementary Table S3**, lymph node metastasis was significantly associated with positive/suspicious clinical lymph node status (*p* = 0.002), increased tumor diameter (*p* = 0.029), higher sVEGF-D levels (*p* = 0.027), greater depth of stromal invasion (*p* = 0.024), and the presence of vascular (*p* = 0.012) and perineural (*p* = 0.005) invasion.

### sVEGF-D Represents an Independent Marker of Lymph Node Involvement

As described above in the *Study Design*, three different models for preoperative prediction of lymph node involvement were built using logistic regression (**Table 4**).

**Table 4:** Logistic regression model estimates for association with prediction of lymph node in cohort A.

Variables	LN clinical evaluation (base model)		Clinical variables (clinical model)		Clinical + sVEGF-D (extended model)		Shrunk coefficients^a^
	OR (95%CI)	*p*-value	OR (95%CI)	*p*-value	OR (95%CI)	*p*-value	
sVEGF-D levels							
(pg/ml), log2 scale	–	–	–	–	4.63 (1.10–19.47)	0.037	3.11
Clinical lymph node status							
Positive *vs*. negative	5.24 (1.86–14.71)	0.002	4.48 (1.46–13.69)	0.009	3.82 (1.07–13.60)	0.039	2.62
Tumor diameter (mm)	–	–	1.03 (1.00–1.06)	0.078	1.04 (1.00–1.08)	0.057	1.03
Tumor grade on biopsy							
G2–G3 *vs*. G1	–	–	2.94 (0.94–9.26)	0.065	3.84 (1.06–13.96)	0.041	2.55
No. of patients	71		70		61		
C-index_adj_ (95%CI)	0.695 (0.565–0.803)		0.743 (0.622–0.882)		0.788 (0.637–0.924)		

Results are reported as odds ratio (OR) and 95% confidence interval (CI). C-index was adjusted for optimism using bootstrap. 95% CI was computed via bootstrap.

LN, lymph node; sVEGF-D, serum vascular endothelial growth factor D; C-index_adj_, adjusted C-index.

a Coefficients computed using penalized maximum likelihood for the extended model.

The base model, including only the clinical/radiological lymph node status, showed a moderate adjusted C-index (C-index_adj_) of 0.70 (95%CI = 0.57–0.80); the clinical model, including the clinicopathological characteristics available before surgery (e.g., tumor diameter, tumor grade evaluated on biopsy, and clinical/radiological lymph node status), showed good discrimination, with C-index_adj_ of 0.74 (95%CI = 0.62–0.88), and good calibration (scaled Brier score = 0.23) (**Supplementary Figure S2A**). The extended model, built by adding the sVEGF-D level to the clinical model, resulted in a significant improvement of the fit (likelihood ratio test: $\chi_{1}^{2}=4.35,$ *p* = 0.037), suggesting an independent association between sVEGF-D and lymph node involvement. Both discrimination and calibration showed a slight improvement, with higher C-index_adj_ (0.79, 95%CI = 0.64–0.92) and scaled Brier score (0.34) (**Supplementary Figure S2B**).

### sVEGF-D Confirms Its Predictive Role in an Independent VSCC Cohort

The extended model applied to cohort B (validation set) showed a reasonably high C-index (0.73, 95%CI = 0.54–0.87). Comparison of the probabilities predicted by the extended model between the two groups of patients divided according to the presence of positive lymph nodes showed good separation with a discrimination slope of 0.78 and an acceptable agreement between the observed and predicted proportions of lymph node metastasis (Hosmer–Lemeshow test, *p* = 0.08) (**Supplementary Figure S3**).

Finally, we evaluated the performance of the extended model on the entire cohort of patients (A + B) in comparison with the base model. The model with sVEGF-D showed a significantly superior performance compared to the clinical/radiological evaluation of the lymph nodes (ROC-AUCs of 0.792 and 0.685, respectively, *p* = 0.008) (**Table 5**). At the optimal cutoff value (Youden), the specificity, sensitivity, positive predictive value (PPV), negative predictive value (NPV), false-negative rate (FNR), and false-positive rate (FPR) of the extended model were 83.0%, 74.4%, 76.3%, 80.4%, 25.6%, and 17.0%, respectively.

**Table 5:** Metrics assessing the performance of the proposed methods for the prediction of lymph node metastasis.

Metrics (95%CI)	LN clinical evaluation	Extended model
AUC	0.685 (0.602–0.768)	0.792 (0.700–0.884)
Specificity (%)	72.2 (61.1–81.9)	83.9 (62.5–92.9)
Sensitivity (%)	64.8 (51.9–77.8)	74.4 (58.1–90.7)
Accuracy (%)	69.0 (61.1–77.0)	78.8 (70.7–85.9)
PPV (%)	63.8 (54.2–73.8)	76.7 (63.6–88.9)
NPV (%)	73.5 (65.8–81.5)	80.4 (72.1–90.9)
FNR (%)	35.2 (22.2–48.1)	25.6 (9.3–41.9)
FPR (%)	27.8 (18.1–38.9)	16.1 (7.1–37.5)
*p*-value (DeLong test)	0.008	

Comparison was made between the performance of the extended model and the base model (LN clinical evaluation) in the whole cohort (A and B). All 95%CIs were calculated using bootstrap (B = 2,000).

LN, lymph node; AUC, area under the curve; PPV, positive predictive value; NPV, negative predictive value; FNR, false-negative rate; FPR, false-positive rate.

## Discussion

Lymph node status is the most important prognostic factor for patients with VSCC, and failure to remove metastatic lymph nodes has serious consequences due to its high mortality (26). On the other hand, removal of lymph nodes can cause both short- and long-term serious side effects (8). For these reasons, a diagnostic test with an optimal sensitivity and specificity would be needed to safely avoid lymphadenectomy. Special attention seems to be focused on high-performance imaging modalities in the prediction of lymph node status, such as ultrasound (27, 28) and ^18^F-FDG-PET/CT (PET) (29–31), in order to better select cN0 patients and identify before surgery those with nodal metastases, even with not palpable nodes. However, to date, imaging cannot diagnose small metastases prior to surgery when their size is below the resolution limits of the available imaging techniques (32).

In this context, circulating biomarkers could offer a valid contribution in the design of a predictive algorithm. For several different tumors such as breast (13), colon (14), ovarian (15), endometrial (16), cervical (33), bladder (34), thyroid (35), gastric (36), esophageal (37), and gallbladder (38) cancers, a strong association between an increased expression of VEGF-D and the presence of lymph node metastases has already been demonstrated by IHC (13–16, 33) or at serum level (34–38); however, investigations on this biomarker regarding VSCC are lacking. To the best of our knowledge, this is the first study investigating the role of sVEGF in VSCC.

In the present study, our findings indicated that an increased serum VEGF-D level is an independent predictor for the presence of metastatic disease in the lymph nodes and an independent risk factor of poor outcomes in terms of both DSS and PFS. In addition, a predictive model adding sVEGF-D to other clinicopathological parameters significantly improved the prediction of nodal metastasis.

Higher levels of sVEGF-D in cancer patients compared to healthy controls or benign pathologies have been detected for some tumor types (36–38) and not for others (34, 35), but regardless of this, a positive correlation with lymph node metastasis has been demonstrated in all studies.

VEGF-C and VEGF-D are key stimulators of both angiogenesis, *via* the activation of VEGFR-2, and lymphangiogenesis, *via* the activation of VEGFR-3, constitutively expressed by lymphatic endothelial cells, promoting growth and remodeling of lymphatic vessels (10, 39, 40). In animal models, VEGF-D-driven lymphatic metastasis has been attributed to both the growth of new lymphatics adjacent to the tumor and the enlargement of preexisting collecting lymphatic vessels (41). In addition to VEGF-D and VEGF-C, VEGF-A, fibroblast growth factor-2, and hepatocyte growth factor have been reported to exert lymphangiogenic activity either directly or indirectly, mediated by the VEGF-C/VEGF-D/VEGFR-3 signaling pathway (42–44).

Tumor spread through the lymphatics is known to be a negative prognosticator, but the molecular mechanisms of VEGF-D as an independent prognostic factor in many types of cancers are still unclear (13–16, 38). One possible hypothesis is that VEGF-D is a mitogenic and morphogenic effector of the proto-oncogene c-Fos and, consequently, may be involved in c-Fos-induced tumor transformation and progression (45, 46).

We found, for the first time, that high circulating levels of sVEGF-D correlate with lymph node metastasis and advanced stage in VSCC patients, as already shown for other malignancies (34–38). On the other hand, the tissue expressions of VEGF-D and VEGFR-3 by IHC did not correlate with any clinicopathological characteristics, as demonstrated by a previous study showing a positive IHC staining for VEGF-D and VEGFR-3 in 100% and 90% of VSCC cases, respectively, and no correlation with the presence of lymph node metastases (17).

According to previous literature (47–49), our study showed that clinical lymph node status, tumor diameter, vascular and perineural invasion, and depth of invasion significantly correlated with inguinofemoral lymph node metastasis. With the aim of providing a useful tool for a tailored surgical approach, we focused our attention on the variables available before surgery: tumor diameter, tumor grade evaluated on biopsy, clinical lymph node status, and sVEGF-D level. The extended model based on these four covariates showed good performance, demonstrated by the C-index of discrimination equal to 0.788. Notably, the performance of the extended model was significantly superior compared to that of the clinical model including only clinical predictors, but excluding sVEGF-D, and to that of the base model based on clinical/radiological evaluation of lymph nodes. The results, validated on an external independent cohort of VSCC patients, confirmed the ability of the extended model to classify patients with respect to lymph node positivity. It is well known that the performance of a predictive model is always lower in the validation group compared to that in the cohort of samples used for its development. The slight difference in the C-index values between our training and validation sets could be explained by the non-homogeneity of the two cohorts as they differed in the surgical approach used to assess lymph nodal status.

Only one previous study proposed two predictive models for groin node metastases based on four parameters (depth of infiltration, grade of differentiation, tumor diameter, and EGFR), but only the first two were independent predictors (50). The two models have not been validated in independent cohorts of patients and their performance is unclear.

Our study provided encouraging preliminary evidence for further investigations on sVEGF-D in association with other clinicopathological variables available preoperatively in the prediction of lymph node metastasis in patients with VSCC. The limits of the present study, which can be mainly attributed to the rarity of this tumor, are mainly related to the relatively small number of cases, the retrospective design, and the prolonged time interval of enrollment, during which many things have changed. The most important evolution concerned the improvement of imaging performance in the prediction of lymph node status due to technological development, the availability of more experienced examiners, and the increased knowledge of this rare pathology obtained by dedicated studies. Currently, imaging provides higher NPV, which in fact is the favored predictive driver since failing to recognize a metastasis and missing the surgical removal could significantly impair prognosis. On the other hand, sVEGF-D could potentially support a higher PPV with other diagnostic tools, being associated with the presence of lymph node metastasis.

In conclusion, it will be essential to validate these retrospective results in larger multicenter prospective studies to identify a threshold value that can achieve FNR and NPV values in accordance with the guidelines of the Gynecologic Cancer Group of the European Organization for Research and Treatment of Cancer (EORTC) (5) and to update the sVEGF-D predictive model integrated with current high-performance imaging methods.

## Data Availability Statement

The raw data supporting the conclusions of this article will be made available by the authors, without undue reservation.

## Ethics Statement

The studies involving human participants were reviewed and approved by Comitato Etico di Brescia, ASST Spedali Civili, Brescia, Italy, and Comitato Etico Policlinico Universitario “A. Gemelli” IRCCS—Università Cattolica del Sacro Cuore, Rome, Italy. The patients/participants provided written informed consent to participate in this study.

## Author Contributions

AR, AG, and FO conceived and designed the study. AR and LA performed the experiments. PA and SC executed the statistical analysis. AO, EB, LZ, CR, and DG collected data. FO, ES, EB, FF, GG, and GS contributed to the analyses and interpretation of data. AR and SC wrote the first drafts of the manuscript. All authors contributed to the article and approved the submitted version.

## Funding

AG and FO were supported by local research grants from the University of Brescia. SC was supported by a research grant from the Italian Ministry of Education, University and Research (PRIN project no. 20178S4EK9).

## Conflict of Interest

The authors declare that the research was conducted in the absence of any commercial or financial relationships that could be construed as a potential conflict of interest.

The reviewer LT declared a shared affiliation with one of the authors GG to the handling editor at the time of review.

## Publisher’s Note

All claims expressed in this article are solely those of the authors and do not necessarily represent those of their affiliated organizations, or those of the publisher, the editors and the reviewers. Any product that may be evaluated in this article, or claim that may be made by its manufacturer, is not guaranteed or endorsed by the publisher.

## Supplementary Material

Supplementary Table 1 Protein expression of VEGF-D and VEGFR-3 on vulvar cancer tissues and correlation with clinicopathological characteristics (49 VSCC patients from Cohort A).

Supplementary Table 2 Univariable survival analysis for both disease specific survival (DSS) and progression free survival (PFS) on VSCC patients from Cohort A.

Supplementary Table 3 Clinicopathologic characteristics of VSCC patients from Cohort A in relation to pathologic lymph node status.

Supplementary Figure 1 Representative immunohistochemical staining for VEGF-D and VEGFR-3 in vulvar invasive squamous cell carcinoma. Magnification 20x.

Supplementary Figure 2 Calibration curves for prediction models. **(A)** Calibration curve for the *clinical model.* **(B)** Calibration curve for the *extended model.*

Supplementary Figure 3 Calibration curve for the *penalized extended model* applied to the validation cohort B.
