# Supplementary material for: VEGF-D Serum Level as a Potential Predictor of Lymph Node Metastasis and Prognosis in Vulvar Squamous Cell Carcinoma Patients
Source: Front Oncol. 2022 Apr 8;12:818613. doi: 10.3389/fonc.2022.818613 (PMC9026339; doi:10.3389/fonc.2022.818613)
Supplement: Supplementary Table 1 — Protein expression of VEGF-D and VEGFR-3 on vulvar cancer tissues and correlation with clinicopathological characteristics (49 VSCC patients from Cohort A). [file Table_1.docx]

**VEGF-D serum level as a potential predictor of lymph node metastasis and prognosis in vulvar squamous cell carcinoma patients**

**Table S1** Protein expression of VEGF-D and VEGFR-3 on vulvar cancer tissues and correlation with clinicopathological characteristics (49 VSCC patients from Cohort A).

|  |  | **VEGF-D score** | |  |  | **VEGFR-3 score** | |  | |
| --- | --- | --- | --- | --- | --- | --- | --- | --- | --- |
| **Clinical characteristics** | | **0-1** | **2-3** | **p-value^a^** |  | **0-1** | **2-3** | **p-value^a^** | |
|  | **Levels (n.)** | **n. (%)** | **n. (%)** |  |  | **n. (%)** | **n. (%)** |  |  |
| **Age (years)** |  |  |  | 0.921 |  |  |  | 0.195 |  |
|  | **<72 (24)** | 8 (50%) | 16 (48%) |  |  | 15 (58%) | 9 (39%) |  |  |
|  | **≥72 (25)** | 8 (50%) | 17 (52%) |  |  | 11 (42%) | 14 (61%) |  |  |
| **FIGO stage** |  |  |  | 0.253 |  |  |  | 0.283 |  |
|  | **I-II (28)** | 11 (69%) | 17 (52%) |  |  | 13 (50%) | 15 (65%) |  |  |
|  | **III-IV (21)** | 5 (31%) | 16 (48%) |  |  | 13 (50%) | 8 (35%) |  |  |
| **Tumor grade** |  |  |  | 0.080 |  |  |  | 0.212 |  |
|  | **G1 (12)** | 6 (37%) | 6 (18%) |  |  | 9 (35%) | 3 (13%) |  |  |
|  | **G2 (30)** | 10 (63%) | 20 (61%) |  |  | 14 (54%) | 16 (70%) |  |  |
|  | **G3 (7)** | 0 (0%) | 7 (21%) |  |  | 3 (11%) | 4 (17%) |  |  |
| **Tumor diameter (mm)** | |  |  | 0.769 |  |  |  | 0.408 |  |
|  | **≤20 (11)** | 3 (19%) | 8 (24%) |  |  | 7 (27%) | 4 (17%) |  |  |
|  | **21-40 (27)** | 10 (62%) | 17 (52%) |  |  | 15 (58%) | 12 (52%) |  |  |
|  | **>40 (11)** | 3 (19%) | 8 (24%) |  |  | 4 (15%) | 7 (30%) |  |  |
| **Depth of invasion (mm)** | |  |  | 0.838 |  |  |  | 0.753 |  |
|  | **≤8 (25)** | 8 (50%) | 17 (53%) |  |  | 13 (50%) | 12 (55%9 |  |  |
|  | **>8 (23)** | 8 (50%) | 15 (47%) |  |  | 13 (50%) | 10 (45%) |  |  |
| **Vascular invasion** | |  |  | 0.773 |  |  |  | 0.717 |  |
|  | **No (35)** | 11 (69%) | 24 (73%) |  |  | 18 (69%) | 17 (74%) |  |  |
|  | **Yes (14)** | 5 (31%) | 9 (27%) |  |  | 8 (31%) | 6 (26%) |  |  |
| **Perineural invasion** | |  |  | 0.117 |  |  |  | 0.721 |  |
|  | **No (29)** | 12 (75%) | 17 (52%) |  |  | 16 (62%) | 13 (57%) |  |  |
|  | **Yes (20)** | 4 (25%) | 16 (48%) |  |  | 10 (38%) | 19 (43%) |  |  |
| **Lymph node metastasis** | |  |  | 0.253 |  |  |  | 0.283 |  |
|  | **No (28)** | 11 (69%) | 17 (52%) |  |  | 13 (50%) | 15 (65%) |  |  |
|  | **Yes (21)** | 5 (31%) | 16 (48%) |  |  | 13 (50%) | 8 (35%) |  |  |
| **Recurrence** |  |  |  | 0.358 |  |  |  | 0.681 |  |
|  | **No (22)** | 6 (37%) | 16 (52%) |  |  | 11 (44%) | 11 (50%) |  |  |
|  | **Yes (25)**  **Unknown** **(2)** | 10 (63%)  0 | 15 (48%)  2 |  |  | 14 (56%)  1 | 11 (50%)  1 |  |  |

^a^ Pearson's Chi-squared test
