# Supplementary material for: VEGF-D Serum Level as a Potential Predictor of Lymph Node Metastasis and Prognosis in Vulvar Squamous Cell Carcinoma Patients
Source: Front Oncol. 2022 Apr 8;12:818613. doi: 10.3389/fonc.2022.818613 (PMC9026339; doi:10.3389/fonc.2022.818613)
Supplement: Supplementary Table 2 — Univariable survival analysis for both disease specific survival (DSS) and progression free survival (PFS) on VSCC patients from Cohort A. [file Table_2.docx]

**VEGF-D serum level as a potential predictor of lymph node metastasis and prognosis in vulvar squamous cell carcinoma patients**

**Table S2** Univariable survival analysis for both disease specific survival (DSS) and progression free survival (PFS) on VSCC patients from Cohort A.

|  |  | **DSS** | |  | **PFS** | |
| --- | --- | --- | --- | --- | --- | --- |
| **Variables** | **N.** | **HR (95% CI)** | **p-value** |  | **HR (95% CI)** | **p-value** |
|  |  |  |  |  |  |  |
|  |  |  |  |  |  |  |
| **Figo stage** |  |  |  |  |  |  |
| I-II | 46 | 1 (-) | - |  | 1 (-) | - |
| III-IV | 33 | 9.43 (3.81-23.34) | **<0.01** |  | 3.20 (1.72-5.98) | **<0.01** |
| **Tumor grade** |  |  |  |  |  |  |
| G1 | 17 | 1 (-) | - |  | 1 (-) | - |
| G2-G3 | 62 | 1.80 (0.69-4.72) | 0.23 |  | 0.96 (1.04-0.62) | 0.38 |
| **Vascular invasion** |  |  |  |  |  |  |
| Absent | 56 | 1 (-) | - |  | 1 (-) | - |
| Present | 23 | 2.55 (1.23-5.26) | **0.01** |  | 1.95 (1.03-3.69) | **0.04** |
| **Perineural invasion** |  |  |  |  |  |  |
| Absent | 48 | 1 (-) | - |  | 1 (-) | - |
| Present | 30 | 2.17 (1.06-4.47) | **0.03** |  | 1.59 (0.85-2.97) | 0.15 |
| **Lymph node metastasis** |  |  |  |  |  |  |
| No | 46 | 1 (-) | - |  | 1 (-) | - |
| Yes | 33 | 9.43 (3.81-23.34) | **<0.01** |  | 3.20 (1.72-5.98) | **<0.01** |
|  |  |  |  |  |  |  |
| **sVEGFD (log scale)** | 62 | 2.70 (1.21-6.03) | **0.02** |  | 2.15 (1.05-4.40) | **0.036** |

Significant p-values are indicated by bold font.
