# Supplementary material for: VEGF-D Serum Level as a Potential Predictor of Lymph Node Metastasis and Prognosis in Vulvar Squamous Cell Carcinoma Patients
Source: Front Oncol. 2022 Apr 8;12:818613. doi: 10.3389/fonc.2022.818613 (PMC9026339; doi:10.3389/fonc.2022.818613)
Supplement: Supplementary Table 3 — Clinicopathologic characteristics of VSCC patients from Cohort A in relation to pathologic lymph node status. [file Table_3.docx]

**VEGF-D serum level as a potential predictor of lymph node metastasis and prognosis in vulvar squamous cell carcinoma patients**

**Table S3** Clinicopathologic characteristics of VSCC patients from Cohort A in relation to pathologic lymph node status.

|  | **Pathologic lymph node status** | |  |  |
| --- | --- | --- | --- | --- |
| **Characteristics** | **Negative (N=46)** | **Positive (N=34)** | **Total (N=80)** | **p-value** |
| **Age (years)** |  |  |  | 0.959^a^ |
| Mean (SD) | 70.3 (11.5) | 70.1 (12.0) | 70.2 (11.7) |  |
| Median (Q1-Q3) | 72.5 (63.0-79.0) | 69.0 (62.5-77.5) | 70.5 (63.0-79.0) |  |
| **Clinical lymph node status** |  |  |  | **0.002^b^** |
| N-Missing | 4 | 5 | 9 |  |
| negative | 32 (76.2%) | 11 (37.9%) | 43 (60.6%) |  |
| positive | 10 (23.8%) | 18 (62.1%) | 28 (39.4%) |  |
| **Tumor grade from biopsy** |  |  |  | 0.104^b^ |
| N-Missing | 1 | 1 | 2 |  |
| G1 | 21 (46.7%) | 9 (27.3%) | 30 (38.5%) |  |
| G2-G3 | 24 (53.3%) | 24 (72.7%) | 48 (61.5%) |  |
| **Tumor diameter (mm)** |  |  |  | **0.029^b^** |
| ≤20 | 12 (26.1%) | 7 (20.6%) | 19 (23.8%) |  |
| 21-40 | 26 (56.5%) | 12 (35.3%) | 38 (47.5%) |  |
| >40 | 8 (17.4%) | 15 (44.1%) | 23 (28.8%) |  |
| **Log_2_ sVEGF-D** |  |  |  | **0.027^a^** |
| N-Missing | 10 | 8 | 18 |  |
| Mean (SD) | 8.71 (0.48) | 9.00 (0.51) | 8.83 (0.51) |  |
| Median (Q1-Q3) | 8.72 (8.49-9.08) | 8.90 (8.68-9.38) | 8.83 (8.55-9.16) |  |
| **Depth of invasion (mm) ^#^** |  |  |  | **0.024^b^** |
| N-Missing | 0 | 1 | 1 |  |
| ≤8 | 29 (63.0%) | 12 (36.4%) | 41 (51.9%) |  |
| >8 | 17 (37.0%) | 21 (63.6%) | 38 (48.1%) |  |
| **Vascular invasion ^#^** |  |  |  | **0.012^b^** |
| Absent | 38 (82.6%) | 19 (55.9%) | 57 (71.2%) |  |
| Present | 8 (17.4%) | 15 (44.1%) | 23 (28.8%) |  |
| **Perineural invasion ^#^** |  |  |  | **0.005^b^** |
| N-Missing | 1 | 0 | 1 |  |
| Absent | 34 (75.6%) | 15 (44.1%) | 49 (62.0%) |  |
| Present | 11 (24.4%) | 19 (55.9%) | 30 (38.0%) |  |
| **Surgical margins ^#^** |  |  |  | 0.771^b^ |
| N-Missing | 0 | 1 | 1 |  |
| Negative | 37 (80.4%) | 28 (84.8%) | 65 (82.3%) |  |
| Positive | 9 (19.6%) | 5 (15.2%) | 14 (17.7%) |  |
| **Tumor grade ^#^** |  |  |  | 0.114^b^ |
| G1 | 13 (28.3%) | 5 (14.7%) | 18 (22.5%) |  |
| G2 | 29 (63.0%) | 21 (61.8%) | 50 (62.5%) |  |
| G3 | 4 (8.7%) | 8 (23.5%) | 12 (15.0%) |  |

# Obtained from surgical specimens.

**^a^**Linear Model ; **^b^**Pearson’s Chi-squared test with simulated p-value (based on 2000 replicates).

Q1-Q3, first and third quartile respectively. Significant p-values are indicated by bold font.
