# Supplementary material for: VEGF-D Serum Level as a Potential Predictor of Lymph Node Metastasis and Prognosis in Vulvar Squamous Cell Carcinoma Patients
Source: Front Oncol. 2022 Apr 8;12:818613. doi: 10.3389/fonc.2022.818613 (PMC9026339; doi:10.3389/fonc.2022.818613)
Supplement: Supplementary Figure 1 — Representative immunohistochemical staining for VEGF-D and VEGFR-3 in vulvar invasive squamous cell carcinoma. Magnification 20x. [file DataSheet_1.pdf]

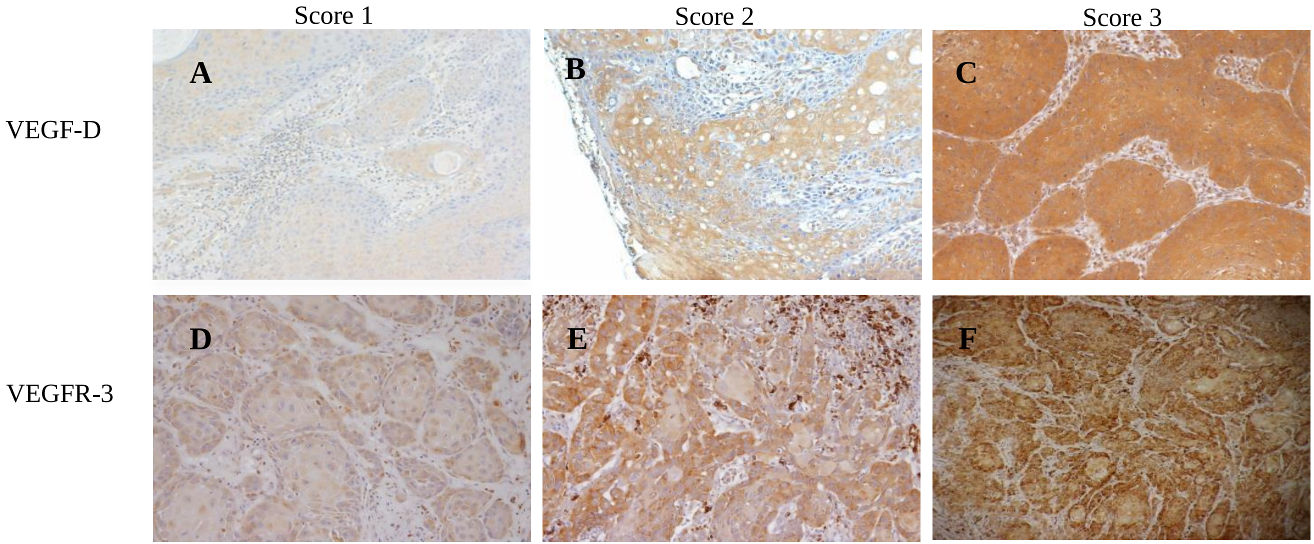

Figure S1. Representative immunohistochemical staining for VEGF-D and VEGFR-3 in vulvar invasive squamous cell carcinoma. Magnification 20x. VEGF-D positive staining was scored 1+ (A), 2+ (B) and 3+ (C) in 24.5%, 53.1% and 14.3% of VSCC primary tissues, respectively. VEGFR-3 positive staining was scored 1+ (D), 2+ (E) and 3+ (F) in 46.9%, 44.9% and 2.0% of VSCC primary tissues, respectively.
