# Supplementary material for: VEGF-D Serum Level as a Potential Predictor of Lymph Node Metastasis and Prognosis in Vulvar Squamous Cell Carcinoma Patients
Source: Front Oncol. 2022 Apr 8;12:818613. doi: 10.3389/fonc.2022.818613 (PMC9026339; doi:10.3389/fonc.2022.818613)
Supplement: Supplementary Figure 2 — Calibration curves for prediction models. (A) Calibration curve for the clinical model. (B) Calibration curve for the extended model. [file DataSheet_2.pdf]

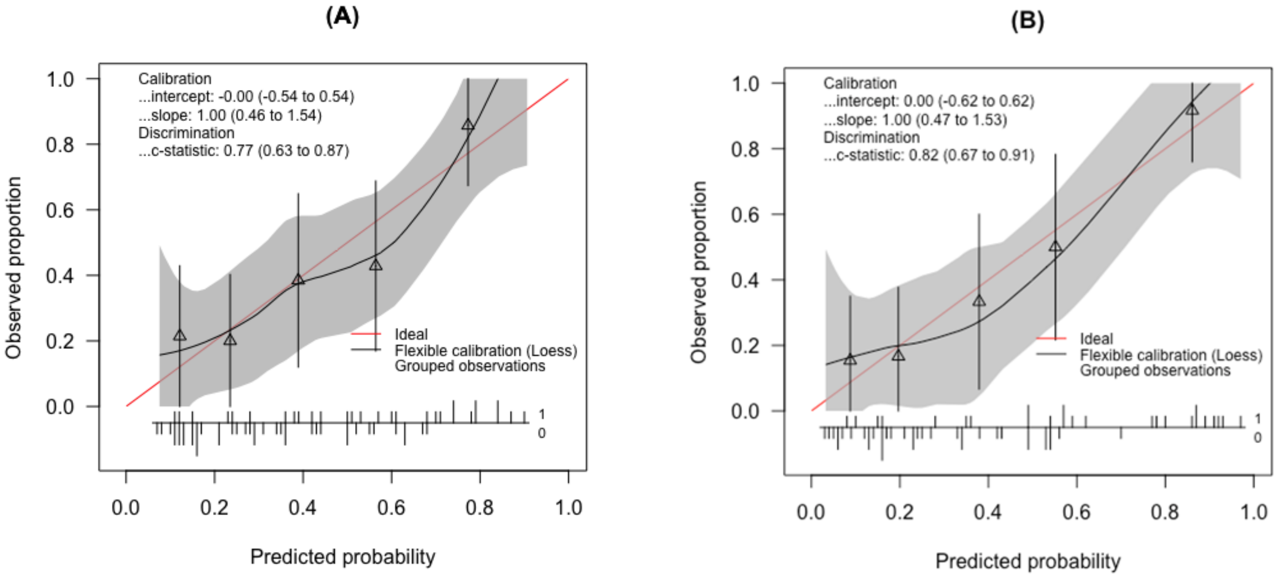

Figure S2. Calibration curves for prediction models. (A) Calibration curve for the clinical model. The Hosmer-Lemeshow test (df=3) had a p-value of 0.41, scaled Brier score=0.23, unadjusted C-index=0.77. (B) Calibration curve for the extended model. The Hosmer-Lemeshow test (df=3) had a p-value of 0.72, scaled Brier score=0.34, unadjusted C-index=0.82. The red line represents the ideal reference line where the predicted probabilities equal the observed ones. Triangles represents average probabilities for grouped observations based on quantiles. Solid black line represents smoothed calibration lines with corresponding 95% confidence interval (gray band).
