# Supplementary material for: VEGF-D Serum Level as a Potential Predictor of Lymph Node Metastasis and Prognosis in Vulvar Squamous Cell Carcinoma Patients
Source: Front Oncol. 2022 Apr 8;12:818613. doi: 10.3389/fonc.2022.818613 (PMC9026339; doi:10.3389/fonc.2022.818613)
Supplement: Supplementary Figure 3 — Calibration curve for the penalized extended model applied to the validation cohort B. [file DataSheet_3.pdf]

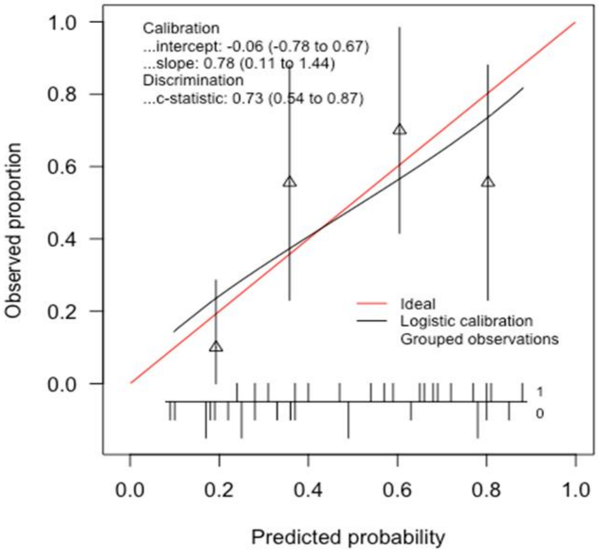

Supplementary Figure S3. Calibration curve for the penalized extended model applied to the validation cohort B. The red line represents the ideal reference line where the predicted probabilities equal the observed ones. Triangles represents average probabilities for grouped observations based on quantiles.
